# 肺癌术后短期中链甘油三酯饮食临床效果的前瞻性随机研究

**DOI:** 10.3779/j.issn.1009-3419.2016.12.04

**Published:** 2016-12-20

**Authors:** 娜 杜, 志勇 饶, 国卫 车, 雪梅 黄, 艳丽 戢, 明铭 王, 梅 杨, 伦旭 刘

**Affiliations:** 1 610041 成都，四川大学华西医院胸外科 Department of Thoracic Surgery, West China Hospital, Sichuan University, Chengdu 610041, China; 2 610041 成都，四川大学华西医院营养科 Department of Nutritional Medicine, West China Hospital, Sichuan University, Chengdu 610041, China

**Keywords:** 肺肿瘤, 中链甘油三酯, 胃肠功能, 快速康复, Lung neoplasms, Medium chain triglyceride diet, Gastrointestinal function fast recovered, Fast track surgery

## Abstract

**背景与目的:**

中链甘油三酯(medium chain triglyceride, MCT)饮食有助于外科手术患者快速康复，本研究将短期MCT食谱应用于肺癌患者术后早期饮食，探讨肺癌患者术后应用MCT的临床效果。

**方法:**

2015年12月-2016年3月四川大学华西胸外科单个医疗组肺癌切除术患者纳入研究患者117例，随机分为MCT组(62例)和常规饮食组(routine diet group, RDG)(55例)。分析两组患者术后白蛋白、肛门排气时间、胸腔闭式引流量及胸腔闭式引流管留置时间、术后住院日、住院费用。

**结果:**

MCT组患者术后肛门排气时间[(27.87±14.38) h]短于RDG组[(45.18±8.62) h](*P* < 0.001)；术后胸腔引流管留置时间在MCT组[(75.40±48.41) h]少于RDG组[(110.64±94.19) h](*P*=0.025)；术后胸腔引流量在MCT[395mL]组少于RDG组[590mL](*P*=0.027)。术后住院日在MCT组[(5.26±2.96) d)]短于RDG组[(6.73±3.99) d](*P*=0.030)。血浆白蛋白术后MCT组[(37.26±2.70) g/L]高于RDG组[(35.92±3.12) g/L](*P*=0.023)。

**结论:**

肺癌患者术后短期应用MCT饮食有助于改善胃肠功能快速恢复，且缩短术后住院时间。

## Abstract (en)

**Background and objective:**

It has been proven that medium chain triglyceride (MCT) diet was contribute to fast track recovery after surgery in patients with operation. In this study, the benefit of postoperative short term MCT diet support was investigated for non-small cell lung cancer patients who underwent anatomic resection.

**Methods:**

A prospective study was planned with the approval of our institutional review board. 117 lung cancer patients who underwent anatomic resection in a single medical group between December 2015 and March 2016 were randomized. 62 patients were applied a MCT diet. and 55 patients in the control group who were fed with only normal diet, and the serum albumin levels, anus exhaust time, chest drainage, chest drainage time, the hospitalization time of post operation, average hospital cost were analysed.

**Results:**

The mean anus exhaust time in MCT group [(27.87±14.38) h] was significantly shorter than that in RDG group [(45.18±8.62) h] (*P* < 0.001); The mean chest tube drainage time in MCT group [(75.40±48.41) h] were less than that in RDG group [(110.64±94.19) h] (*P*=0.025). The mean chest tube drainage volume in MCT group [395 mL] were less than that in RDG group [590 mL] (*P*=0.027). The hospitalization time of post operation was (5.26±2.96) d in MCT group against (6.73±3.99) d in RGD group (*P*=0.030). The serum albumin levels of postoperative in MCT group [(37.26±2.70) g/L)] was significantly higher than that in RDG group [(35.92±3.12) g/L)] (*P*=0.023).

**Conclusions:**

The results of this study demonstrated that postoperative short term MCT diet is beneficial in postoperative gastrointestinal function fast recovered and shorten the hospitalization time in non-small cell lung cancer patients after surgery.

围手术期充分营养支持是保证手术创伤(如切口)愈合和降低术后并发症的基础^[1]^，而肺癌患者围手术期饮食无统一方案。加速康复外科需要优化围手术期流程^[2]^，以适应新的麻醉和手术流程，目前大家关注最多的是手术和麻醉^[3]^，而忽视了术后管理尤其是饮食。通常做法是进食高蛋白和高脂饮食[以长链脂肪酸为主(long chain triglyceride, LCT)]。但研究发现术后早期进食高动物性蛋白及脂类食物导致乳糜胸及胃肠功能障碍^[4]^。而以中链甘油三酯(medium chain triglyceride, MCT)为主的饮食，对胃肠道的抑制胃排空作用(十二指肠-胃反馈抑制作用)较LCT弱^[5]^，且经肠摄入的MCT不形成乳糜微粒而经淋巴系统转运，可以减少手术患者淋巴管瘘而导致的脂肪丢失和引流液(乳糜液)漏出量^[6]^。因此，肺癌患者术后应用短期MCT饮食是否能够达到降低引流量和促进胃肠功能恢复，从而达到患者快速康复呢？尚没有看到此方面的研究与报道，我们前瞻性分析了117例肺癌术后短期分别应用MCT饮食和常规饮食对术后相关临床资料，初步探讨术后短期MCT饮食临床应用的可行性及不足和优势。

## 1 资料与方法

### 1.1 临床资料

连续分析2015年12月-2016年3月在四川大学华西医院胸外科单个医疗组行肺癌手术(肺叶或肺段)患者151例。纳入标准：①年龄19岁-75岁；②病理学检查诊断为原发性肺癌；③手术方式肺段、肺叶(单叶或双叶)切除术+系统淋巴结清扫术；④临床资料完整且签署知情同意书。排除标准：①病理诊断为转移性肺癌患者或病历资料不完整；②未签署知情同意书的患者；③术前诊断为营养不良或术后在重症监护室监护大于48 h的患者；④术后出血或持续漏气需要再次手术的患者。最终纳入患者117例(图 1)，其中实验组(MCT)组62例，对照组[常规饮食组(routine diet group, RDG)]55例。术后分期采用国际抗癌联盟(Union for International Cancer Control, UICC)(2009)肺癌分期标准。患者临床特征见表 1。

**1:**
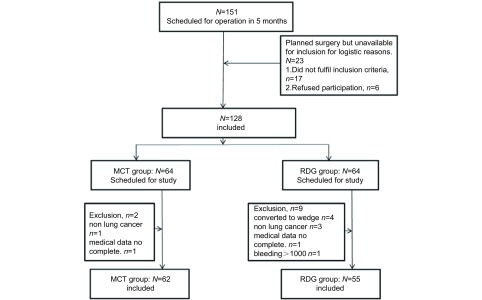
两组患者筛选流程图 Study profile for two group cases. MCT: medium chain triglyceride; RDG: routine diet.

**1:** 两组患者临床特征 The characteristics of the patients

Characteristics		MCT (*n*=62)	RDG (*n*=55)	*P*
Gender	Male	36	33	0.371
	Female	26	22	
Age (yr)	Male	55.72±10.65	57.28±11.30	0.474
	Female	57.33±9.76	55.13±10.38	0.946
Smoking	Yes	40	36	0.900
	No	22	19	
Comorbidity	Yes	39	32	0.460
	No	23	23	
	Hypertension	29	14	0.373
	Diabetes	3	4	
	Coronary heart disease	3	2	
	Hepatitis B	1	2	
	Chronic bronchitis	0	3	
	Emphysema	0	2	
	Hypercholesterolemia	0	1	
	Liver and kidney cyst	2	4	
	Dilated cardiomyopathy	1	0	
Operation	VATS	56	51	0.152
	Open	6	4	
Histology	Adenocarcinoma	48	43	0.627
	Adenosquamous carcinoma	10	10	0.425
	Other	4	2	0.984
TNM stage (2009 UICC)	Ⅰ	48	40	0.217
	Ⅱ	9	4	0.562
	Ⅲ	2	10	0.006
	Ⅳ	3	1	0.851
VATS: video-assisted thoracic surgery; TNM: tumor-node-metastasis; UICC: Union for International Cancer Control.				

### 1.2 方法

手术方法：手术方式应用开胸或单向式胸腔镜肺叶切除法+系统淋巴结清扫^[7]^。系统淋巴结清扫左侧必须清扫第5、6、7、8、9、10组淋巴结，右侧包括第2、3、4、7、8、9、10组淋巴结^[8]^。引流管应用方法：胸腔引流管统一选用扬州市邗江华飞医疗器件厂生产的一次性使用硅橡胶28F和16F引流管，均应用单引流管是将16或28F硅橡胶引流管从第7肋间镜孔经后胸胸壁向上直达胸顶，不需另加侧孔，两组患者术后均应用相同的水封引流瓶，且均不加用负压吸引^[9]^；16号组不加用留置线，28号需应用留置线。

### 1.3 术后饮食管理

#### 1.3.1 MCT食物主要成分

热量(1, 687.6 kcal)，膳食纤维(11.69 g)，蛋白质(62.58 g)，碳水化合物(265.19 g)，MCT(30.0 g)，脂肪(11.67 g)，胆固醇(0 mg)；维生素类：A(445.3 μg)，B1(0.8 mg)，B2(0.63 mg)，C(184.8 mg)，E(9.49 mg)，叶酸(175.76 μg)，烟酸(9.83 mg)；微量元素：钙(883 mg)，磷(1, 052.4 mg)，钠(1 989.87 mg)，钾(1, 850.95 mg)，镁(342.6 mg)，铁(23.47 mg)，锌(10.83 mg)，硒(17.95 μg)，铜(2.7 mg)，锰(7.52 mg)。

#### 1.3.2 术后饮食方案

对照组(RDG)采用常规饮食护理：术后4 h病员神志清楚后可口服适量温开水，无恶心、呕吐不适，6 h-8 h可进少量流质。术后第1天可正常饮食。实验组(LPDG)：术后4 h，神志清楚后口服100 mL温开水，无恶心、呕吐不适，6 h-8 h饮用开胃流质250 mL，术后10 h-12 h口服50 g营养粉，兑温水250 mL，术后第1天-3天，营养科订餐，MCT饮食，可喝水，进食水果。术后第4天恢复正常饮食。

### 1.4 术后处理

气管插管拔管后均鼓励患者咳嗽，必要时刺激患者咳嗽。术后第1天均行胸部照片，若无漏气且每天引流量小于300 mL，肺已复张则拔除引流管^[10]^。术后疼痛处理均应用镇痛泵(5 mg负荷剂量，1.0 mg/h-1.5 mg/h)，均早期促使患者下床活动。必要时应用非甾体类止痛药(泰勒宁或芬必得)。镇痛泵于引流管拔除的同时也一起停止^[10]^。

### 1.5 观察指标

#### 1.5.1 乳糜胸

诊断标准：乳糜试验(+)且每天引流量大于500 mL。

#### 1.5.2 胸腔引流管留置时间及引流量

从手术后安置到拔除时间；术后胸腔总引流量，从胸腔引流管安置到拔除时的总引流量。

#### 1.5.3 术后排气时间

从手术结束到病房患者自诉排气时间。

#### 1.5.4

术后肝肾功能指标为术后第3天检测。

#### 1.5.4 术后住院日

手术当天到出院当天时间(出院当天计算在内，实际上应去除)

#### 1.5.5 住院总费用

住院期间所产生的费用，不包括门诊检查或治疗所产生的费用。

### 1.6 统计学分析

统计分析采用SPSS 16.0软件包，计数资料采用实际例数及百分比表示，正态分布计量资料采用均数±标准差(Mean±SD)表示，正态分布计量资料比较采用两独立样本的*t*检验；非正态分布计量资料采用中位数(median)表示，非正态分布计量资料采用两独立样本的秩和检验(*Mann-Whitney U*法)。计数资料的比较采用χ^2^检验或确切概率法进行分析。*P* < 0.05为差异有统计学意义。

## 2 结果

### 2.1 两组患者术后临床结果分析

MCT组患者术后胃肠功能恢复时间[(27.87±14.38) h]短于RDG组[(45.18±8.62) h](*P* < 0.001)；术后胸腔引流管留置时间在MCT组[(75.40±48.41) h]少于RDG组[(110.64±94.19) h](*P*=0.025)；术后胸腔引流量在MCT[395 mL]组少于RDG组[590 mL](*P*=0.027)。且术后住院日在MCT组[(5.26±2.96) d)]短于RDG组[(6.73±3.99) d](*P*=0.030)。而乳糜胸发生率和住院平均费用在两组之间均无差异(表 2)。

**2:** 两组患者术后相关临床指标比较 Comparison of laboratory index of correlation between MCT group and RDG group in post operation

	MCT	RDG	*P*
Anus exhaust time (h)	27.87±14.38	45.18±8.62	< 0.001
Median of chest drainage (mL)	395	590	0.027
Chest drainage time (h)	75.40±48.41	110.64±94.19	0.025
Chylothorax	0	0	-
The hospitalizationtime of post operation (d)	5.26±2.96	6.73±3.99	0.030
Average hospital cost (￥)	47, 660.70±9, 883.25	51, 147.99±11, 620.67	0.097

### 2.2 两组患者术后生化相关指标比较

两组患者血红蛋白(hemoglobin, Hb)在术后均有降低，但两组患者手术前、后均无统计学差异(*P*=0.602, *P*=0.733)；而手术前、后血肌酐在两组均无统计学差异(*P*=0.515, *P*=0.595)。血浆白蛋白术前在MCT组[(43.44±2.55) g/L]与RDG组[(42.53±2.77) g/L]无统计学差异(*P*=0.084)，而术后MCT组[(37.26±2.70) g/L]显著高于RDG组[(35.92±3.12) g/L](*P*=0.023)(表 3)。

**3:** 两组患者手术前后实验室检查相关指标 Comparison of laboratory index of correlation between MCT group and RDG group in preoperation and post-operation

		MCT	RDG	*P*
Hb (g/L)	Pre	136.41±15.98	135.93±13.11	0.602
	Post	122.05±13.87	122.95±12.13	0.733
Albumin (g/L)	Pre	43.44±2.55	42.53±2.77	0.084
	Post	37.26±2.70	35.92±3.12	0.023
ALT (u/L)	Pre	24.12±13.12	20.16±9.22	0.010
	Post	22.17±15.54	19.60±9.82	0.343
Creatinine	Pre	63.59±15.24	68.33±17.95	0.515
	Post	62.39±15.54	64.21±18.89	0.595
Hb: hemoglobin; ALT: alanine aminotransferase.				

## 3 讨论

外科患者围手术期营养在消化道手术中的应用，取得了明显的临床效果，体现在降低围手术期并发症和促进患者的术后康复^[11]^。而非消化道手术营养支持，因禁食时间短，一直以来没有相应营养和饮食方案，相关研究也很少。肺癌患者术后饮食随意性比较强，多数患者以高脂和高蛋白为主，以期增加营养，但近期有研究发现术后高蛋白和高脂饮食易致腹胀和乳糜胸，且增加胸腔引流量^[6]^。研究表明：MCT有以下特点：①饱和度高，因此氧化稳定性较好；②脂肪酸碳链短，因此具有更好的亲水性；③进入线粒体无需肉毒碱，因此代谢完全，供能快^[12]^。因此其主要应用人群有^[13]^：外科手术患者、肿瘤患者、糖尿病患者、淋巴循环紊乱患者、食物过敏患者、营养不良患者。根据以上MCT饮食特点，本研究探讨肺癌患者术后短期应用MCT饮食的优势及效果。

麻醉、术中创伤应激和术后镇痛导致的胃肠功能减退或不适均影响胃肠功能恢复，严重时导致腹胀，而摄食的MCT对胃肠道的影响不同于LCT，抑制胃排空作用(十二指肠-胃反馈抑制作用)较弱^[13]^，但刺激胆囊收缩素释放的作用较LCT更强^[14]^。在胃和十二指肠内被脂肪酶分解成甘油和中链脂肪酸，有较好的水溶性，其水解速率是LCT的6倍^[15]^，直接通过小肠毛细血管进入门静脉，然后快速转移到肝内^[16]^。肺癌患者术后短期应用MCT饮食可否促进胃肠功能恢复呢？我们研究表明术后短期MCT饮食明显缩短肛门排气时间，即促进胃肠功能恢复。

研究发现乳糜腹水及乳糜胸是复杂的腹部手术后的常见并发症^[17, 18]^，应首选以MCT替代LCT膳食为主的对症保守治疗^[18, 19]^。由于经肠摄入的MCT不形成乳糜微粒而经淋巴系统转运，可以减少手术患者淋巴管瘘而导致的脂肪的丢失和引流液(乳糜液)漏出量。肺癌患者因清扫纵隔和肺门淋巴结，损伤大量淋巴管道，易形成乳糜胸或增加术后引流量^[20]^，而过多的高脂或蛋白饮食也会增加乳糜胸发生机率和增加胸腔引流量^[21]^，过多的引流会导致胸腔引流管留置时间过长，不利于术后快速康复。MCT组胸腔引流量少于常规饮食组，且引流管留置时间缩短，均证明MCT饮食有助于减少因淋巴管道损伤导致的漏出液过多。同时研究中也发现，MCT饮食没有增加蛋白供应量，而MCT组术后患者血浆白蛋白水平高于RGD组，分析其原因主要是MCT组患者胸腔引流量减少，导致的蛋白损失减少所致。这也说明肺癌患者术后短期MCT饮食不会影响患者的营养状况，反而有助于改善脏器功能。

总之，我们研究表明，肺癌患者术后短期MCT饮食有助于促进胃肠功能的快速恢复、降低胸腔引流量和减少引流管留置时间，从而缩短术后住院时间且不增加患者费用，达到快速康复的目的。但本研究也存在常规饮食组排除患者过多，样本含量小的问题，需要进一步研究。
